# Uniform Error Estimates of the Finite Element Method for the Navier–Stokes Equations in $R^{2}$ with *L*^2^ Initial Data

**DOI:** 10.3390/e25050726

**Published:** 2023-04-27

**Authors:** Shuyan Ren, Kun Wang, Xinlong Feng

**Affiliations:** 1 College of Mathematics and System Sciences, Xinjiang University, Urumqi 830017, China; shuyan_ren_math3@stu.xju.edu.cn (S.R.); fxlmath@xju.edu.cn (X.F.); 2 College of Mathematics and Statistics, Chongqing University, Chongqing 401331, China

**Keywords:** Navier–Stokes equations, finite element method, uniform error estimate, *L*^2^ initial data

## Abstract

In this paper, we study the finite element method of the Navier–Stokes equations with the initial data belonging to the $L^{2}$ space for all time t>0. Due to the poor smoothness of the initial data, the solution of the problem is singular, although in the $H^{1}$-norm, when t∈[0,1). Under the uniqueness condition, by applying the integral technique and the estimates in the negative norm, we deduce the uniform-in-time optimal error bounds for the velocity in $H^{1}$-norm and the pressure in $L^{2}$-norm.

## 1. Introduction

In this paper, we consider the error estimates of the mixed finite element approximation to the time-dependent Navier–Stokes equations with nonsmooth initial data as follows:

(1) $$\begin{array}{cc} \; & u_{t}-\nu \Delta u+\left(u\cdot \nabla \right)u+\nabla p=f,\;\;\;\mathrm{div}\;u=0,\;\;\;\left(x,t\right)\in \Omega \times R^{+}; \end{array}$$

(2) $$\begin{array}{cc} \; & u\left(x,0\right)=u_{0}{\left(x\right),\;\;x\in \Omega ;\;\;u\left(x,t\right)|}_{\partial \Omega }=0,\;\;\;t\geq 0, \end{array}$$

where Ω is a bounded domain in $R^{2}$ that has a Lipschitz continuous boundary ∂Ω and satisfies the additional condition (**A1**) (see below), ν>0 is the viscosity, $u=u\left(x,t\right)={\left(u_{1}\left(x_{1},x_{2},t\right),u_{2}\left(x_{1},x_{2},t\right)\right)}^{T}$ is the velocity, p=p(x,t) is the pressure, $f=f\left(x,t\right)={\left(f_{1}\left(x_{1},x_{2},t\right),f_{2}\left(x_{1},x_{2},t\right)\right)}^{T}$ is the prescribed body force, and $u_{0}\left(x\right)$ is the initial velocity.

Many works are devoted to the finite element approximation of the Navier–Stokes Equations (1) and (2). The reader is referred to [1,2,3,4,5,6,7,8], for instance. In these classical works, they usually considered the problem under the smooth initial data condition ($u_{0}\left(x\right)\in H_{0}^{1}\left(\Omega \right)$ or $u_{0}\left(x\right)\in H^{2}\left(\Omega \right)\cap H_{0}^{1}\left(\Omega \right)$). There are few papers on the problem with the rough initial data. When $u_{0}\left(x\right)$ only belongs to the $L^{2}\left(\Omega \right)$ space, the solution of the system (1) and (2) is singular, although in the $H^{1}$-norm. Therefore, the classical error analysis technique is not feasible in this case. However, various issues are considered in other references, e.g., see [9,10] for the finite element method of the linear parabolic equations and [11,12,13,14] for the Navier–Stokes equations. In [11], the stability of the finite element method for the Navier–Stokes equations with the nonsmooth initial data was obtained on the finite time interval. Due to the special character of the spectral operator and using the high-dimensional spectral space when t∈[0,1) and the low-dimensional spectral space when t∈[1,∞), they gave $L^{2}$ error estimates for the velocity of the spectral method [12]. In fact, they applied the two-grid method in analysis. Recently, the $H^{2}$-stability of the first- and second-order fully discrete schemes were investigated in [13,14], respectively, and these analysis techniques were extended to other nonlinear problems, such as the Oldroyd model [15], the natural convection equations [16], and the Boussinesq equations [17]. On the other hand, the long-time analysis for the numerical method is also very significant. The reader is referred to [7,18,19] for more details. However, according to the authors’ best knowledge, error estimates for the Navier–Stokes equation with initial data belonging to $L^{2}\left(\Omega \right)$ are not available.

In this paper, first, we divide the error of the finite element method into two parts: one part is generated by the approximate linearization problem, and the other part is generated by the approximate nonlinear term. Then, based on the stability of the solution of the problems (1) and (2) with $u_{0}\left(x\right)\in L^{2}\left(\Omega \right)$ given in [12], assuming the given data satisfying the uniqueness condition, and using the integral technique and the estimates in the negative norm to overcome the singularity of the solution on t∈[0,1), we derive the finite element error estimates for the linearized problem, and the error resulting from the approximation of the nonlinear term can also be obtained from the trigonometric inequality.

The paper is organized as follows. In the next section, we will recall some functional settings for the problem. Then, we will introduce the finite element approximation and the stability of finite element solutions in Section 3, and derive the uniform error estimates for the velocity and pressure in Section 4. In Section 5, we show some numerical examples to verify the theoretical predictions. Finally, conclusions are made in Section 6.

## 2. Functional Settings

In this section, we introduce the notation used in what follows. We introduce the Hilbert spaces:

$$\begin{array}{cc} \; & X={\left(H_{0}^{1}\left(\Omega \right)\right)}^{2},\;\;\;Y={\left(L^{2}\left(\Omega \right)\right)}^{2},\;\;\;M=L_{0}^{2}\left(\Omega \right)=\left\{q\in L^{2}\left(\Omega \right);\int_{\Omega }qdx=0\right\}, \end{array}$$

where $\left|\right|\cdot {\left|\right|}_{i}$ is the usual norm of the Sobolev space $H^{i}\left(\Omega \right)$ or ${\left(H^{i}\left(\Omega \right)\right)}^{2}$ for *i* = 1, 2, and (·,·) and |·| is the inner product and norm of $L^{2}\left(\Omega \right)$ or ${\left(L^{2}\left(\Omega \right)\right)}^{2}$, respectively. The scalar product and norm of the spaces $H_{0}^{1}\left(\Omega \right)$ and *X* are given by

$$\begin{array}{c} \left(\left(u,v\right)\right)=\left(\nabla u,\nabla v\right),\;\;\;||u||={\left(\left(u,u\right)\right)}^{1/2}. \end{array}$$

We also define the closed smooth solenoidal vector fields *V* in the norm of *X* and the closed smooth solenoidal vector fields *H* in the norm of *Y* as

$$\begin{array}{c} V=\left\{v\in X;\mathrm{div}v=0\right\},\;\;\;H=\left\{v\in Y;\mathrm{div}v=0,v\cdot n{|}_{\partial \Omega }=0\right\}, \end{array}$$

where *n* is the unit outerward normal vector of the domain boundary and the Stokes operator by A=−PΔ, and the Laplace operator $\tilde{A}=-\Delta$, where *P* is the $L^{2}$-orthogonal projection of *Y* onto H.

To proceed, we need a further assumption concerning Ω:

(**A1**) Assume that Ω is regular in the sense that a unique solution (v,q)∈(X,M) of the Stokes problem

$$\begin{array}{c} {-\nu \Delta v+\nabla q=g,\;\;\;\mathrm{div}\;v=0\;\mathrm{in}\;\Omega ,\;\;\;v|}_{\partial \Omega }=0, \end{array}$$

for any prescribed g∈Y exists and satisfies

$$\begin{array}{c} {\left||v|\right|}_{2}+{\left||q|\right|}_{1}\leq c_{0}\left|g\right|, \end{array}$$

where $c_{0}>0$ is a positive constant. Hereafter, $\kappa ,c,c_{i}>0,\;i=0,1,2,\cdots$ are generic positive constants independent of the mesh size *h*. They are subject to different values in different cases.

(**A1**) implies

(3) $$\begin{array}{cc} \; & {\left|v\right|}^{2}\leq \lambda_{1}^{-1}{\left||v|\right|}^{2}\;\;\forall v\in X,\\ \; & {\left||v|\right|}^{2}\leq \lambda_{1}^{-1}{\left|Av\right|}^{2}{,\;\;\;||v||}_{2}^{2}\leq c{\left|Av\right|}^{2}\;\;\forall v\in D\left(A\right)={\left(H^{2}\left(\Omega \right)\right)}^{2}\cap V, \end{array}$$

where $\lambda_{1}$ is the minimal eigenvalue of the Laplace operator −Δ.

Furthermore, we make the following assumptions on the prescribed data for the problems (1) and (2):

(**A2**) The initial velocity $u_{0}\in H$ and the body force f(x,t) satisfy

$$\begin{array}{c} f,f_{t}\in L^{\infty }\left(R^{+};Y\right)\mathrm{with}\;\;|u_{0}|+\sup{}_{t\geq 0}(|f\left(t\right)|+\tau \left(t\right)|f_{t}\left(t\right)\left|\right)\leq \kappa , \end{array}$$

where τ(t)=min{1,t}.

The continuous bilinear forms a(·,·) on X×X and d(·,·) on X×M are, respectively, defined by

$$\begin{array}{c} a(u,v)=\nu ((u,v))\;\;\;\forall u,v\in X,\;\;\;d(v,q)=(q,\mathrm{div}\;v)\;\;\;\forall v\in X,q\in M. \end{array}$$

Obviously, the bilinear form a(·,·) is continuous and coercive, and the bilinear form d(·,·) is continuous and satisfies the inf-sup condition known as the Ladyzhenskaya–Babu$\overset{ˇ}{s}$ka–Brezzi (LBB) condition [1]: there exists a positive constant $\beta_{0}$ such that

(4) $$\begin{array}{c} \underset{v\in X,v\neq 0}{\sup}\frac{d(v,q)}{∥v∥}\geq \beta_{0}\left|q\right|,\;\forall q\in M. \end{array}$$

with

$$\begin{array}{c} B\left(u,v\right)=\left(u\cdot \nabla \right)v+\frac{1}{2}\left(\mathrm{div}\;u\right)v, \end{array}$$

the trilinear form b(·,·,·) is, for all ∀u,v,w∈X,

$$\begin{array}{cc} b(u,v,w) & =\left(B\left(u,v\right),w\right)=\left(\left(u\cdot \nabla \right)v,w\right)+\frac{1}{2}\left(\left(\mathrm{div}\;u\right)v,w\right)\\ \; & =\frac{1}{2}\left(\left(u\cdot \nabla \right)v,w\right)-\frac{1}{2}\left(\left(u\cdot \nabla \right)w,v\right). \end{array}$$

It holds true that (see [4,20])

(5) $$\begin{array}{cc} \; & b(u,v,w)=-b(u,w,v)\;\;\;\forall u,v,w\in X,\\ \; & \left|b\left(v,u,w\right)\right|+\left|b\left(u,v,w\right)\right|\leq c_{1}{\left(|u\right|}^{1/2}{\left||u|\right|}^{1/2}\left||v|\right| \end{array}$$

(6) $$\begin{array}{cc} \; & \;\;\;+{\left||u||\;|v\right|}^{/1/2}{\left||v|\right|}^{1/2}{)|w|}^{1/2}{\left||w|\right|}^{1/2}\;\;\;\forall u,v,w\in X,\\ \; & \left|b\left(u,v,w\right)\right|+\left|b\left(v,u,w\right)\right|\leq c_{1}{\left(||u||\;|v\right|}^{1/2}{\left|Av\right|}^{1/2} \end{array}$$

(7) $$\begin{array}{cc} \; & \;\;\;+{\left|u\right|}^{1/2}{\left||u|\right|}^{1/2}{\left||v|\right|}^{1/2}{\left|Av\right|}^{1/2})|w|\;\;\;\forall u,w\in X,v\in D\left(A\right), \end{array}$$

(8) $$\begin{array}{cc} \; & |b(u,v,w)|\leq N||u||\;||v||\;||w||\;\;\;\forall u,v,w\in X,\\ \; & \left|b(u,v,w)|+|b(u,w,v)|+|b(w,u,v)\right|\\ \; & \;\;\;\leq \frac{1}{3}c_{1}{\left(|u\right|}^{1/2}{\left|Au\right|}^{1/2}\left|Av\right|+{\left|v\right|}^{1/2}{\left|Av\right|}^{1/2}{\left|Au|)||w|\right|}_{-1} \end{array}$$

(9) $$\begin{array}{cc} \; & \;\;\;+\frac{1}{3}c_{1}{\left||u|\right|}^{1/2}{\left|Au\right|}^{1/2}{\left|Av\right|}^{1/2}{\left||v|\right|}^{1/2}{\left||w|\right|}_{-1}\;\;\;u,v,w\in V, \end{array}$$

where $c_{1}$ is only dependent on the domain Ω. In this notation, the weak formulation of the problems (1) and (2) is as follows: Find (u,p)∈(X,M), such that for all t>0,(v,q)∈(X,M),

(10) $$\begin{array}{cc} \; & \left(u_{t},v\right)+a\left(u,v\right)-d\left(v,p\right)+d\left(u,q\right)+b\left(u,u,v\right)=\left(f,v\right), \end{array}$$

(11) $$\begin{array}{cc} \; & u\left(x,0\right)=u_{0}. \end{array}$$

**Theorem** **1.** *Under the assumptions (* ***A1*** *) and (* ***A2*** *), the problems (1) and (2) admits a unique solution, which satisfies the following bounds for all t>0:*

(12) $$\begin{array}{c} {\left|u\left(t\right)\right|}^{2}+e^{-2\delta_{0}t}\int_{0}^{t}e^{2\delta_{0}\tilde{t}}{\left||u|\right|}^{2}d\tilde{t}\leq \kappa , \end{array}$$

(13) $$\begin{array}{c} {\tau \left(t\right)||u\left(t\right)||}^{2}+e^{-2\delta_{0}t}\int_{0}^{t}e^{2\delta_{0}\tilde{t}}\tau \left(\tilde{t}\right){\left(|Au\right|}^{2}+|u_{\tilde{t}}{|}^{2})d\tilde{t}\leq \kappa , \end{array}$$

(14) $$\begin{array}{c} \tau^{2}{\left(t\right)(|Au\left(t\right)|}^{2}+|u_{t}{\left(t\right)|}^{2})+e^{-2\delta_{0}t}\int_{0}^{t}e^{2\delta_{0}\tilde{t}}\tau^{2}\left(\tilde{t}\right)\left|\right|u_{\tilde{t}}{\left|\right|}^{2}d\tilde{t}\leq \kappa , \end{array}$$

(15) $$\begin{array}{c} \tau^{3}\left(t\right)||u_{t}{\left(t\right)||}^{2}+e^{-2\delta_{0}t}\int_{0}^{t}e^{2\delta_{0}\tilde{t}}\tau^{3}\left(\tilde{t}\right)(|Au_{\tilde{t}}{|}^{2}+|u_{\tilde{t}\tilde{t}}{|}^{2})d\tilde{t}\leq \kappa , \end{array}$$

(16) $$\begin{array}{c} \lim_{t\to \infty }\sup||u\left(t\right)||\leq \nu^{-1}\left|\right|f_{\infty }{\left|\right|}_{-1}, \end{array}$$

(17) $$\begin{array}{c} {\tau \left(t\right)(|p\left(t\right)|}^{2}+\left|\right|u_{t}{\left(t\right)||}_{-1}^{2})+e^{-2\delta_{0}t}\int_{0}^{t}e^{2\delta_{0}\tilde{t}}\left(|\right|u_{\tilde{t}}{\left|\right|}_{-1}^{2}+{\left|p\right|}^{2})d\tilde{t}\leq \kappa , \end{array}$$

(18) $$\begin{array}{c} \tau^{2}{\left(t\right)||p\left(t\right)||}_{1}^{2}+e^{-2\delta_{0}t}\int_{0}^{t}e^{2\delta_{0}\tilde{t}}(\tau \left(\tilde{t}\right){\left||p|\right|}_{1}^{2}+\tau^{2}\left(\tilde{t}\right)|p_{\tilde{t}}{|}^{2}+\tau^{3}\left(\tilde{t}\right)\left|\right|p_{\tilde{t}}{\left|\right|}_{1}^{2})d\tilde{t}\leq \kappa , \end{array}$$

*where $0<\delta_{0}<\frac{1}{2}\nu \lambda_{1}$, $f_{\infty }\left(x\right)=\lim_{t\to \infty }f\left(x,t\right)$ and $\left|\right|f_{\infty }{\left|\right|}_{-1}=\underset{v\in X,v\neq 0}{\sup}\frac{\left(f_{\infty },v\right)}{\left||v|\right|}.$*

**Proof.**  The existence and uniqueness of the solution are provided in, e.g., Section 3 of Chapter II in [20,21]. For (12)–(15), the reader is referred to Theorem 2.1 in [12]. We only need to prove the other inequalities.

Taking $\left(v,q\right)=e^{2\delta_{0}t}\left(u,p\right)$ in (10) and using (5), we have

(19) $$\begin{array}{c} \frac{1}{2}\frac{d}{dt}(e^{2\delta_{0}t}{\left|u\left(t\right)\right|}^{2})+\nu e^{2\delta_{0}t}{\left||u|\right|}^{2}=e^{2\delta_{0}t}\left(f,u\right)+\delta_{0}e^{2\delta_{0}t}{\left|u\right|}^{2}. \end{array}$$

Integrating (19) with respect to the time from 0 to *t* and multiplying by $e^{-2\delta_{0}t}$, we arrive at

(20) $$\begin{array}{cc} \; & {\left|u\left(t\right)\right|}^{2}+2\nu e^{-2\delta_{0}t}\int_{0}^{t}e^{2\delta_{0}\tilde{t}}{\left||u|\right|}^{2}d\tilde{t}\\ = & 2\delta_{0}e^{-2\delta_{0}t}\int_{0}^{t}e^{2\delta_{0}\tilde{t}}{\left|u\right|}^{2}d\tilde{t}+2e^{-2\delta_{0}t}\int_{0}^{t}e^{2\delta_{0}\tilde{t}}\left(f,u\right)d\tilde{t}. \end{array}$$

Letting t→∞ and using the L’Hospital rule, we find that

$$\begin{array}{c} \nu \lim_{t\to \infty }{\sup||u\left(t\right)||}^{2}\leq \lim_{t\to \infty }\sup\left(f\left(t\right),u\left(t\right)\right)\leq \left|\right|f_{\infty }{\left|\right|}_{-1}\lim_{t\to \infty }\sup||u\left(t\right)||, \end{array}$$

which implies (16).

Applying the Stokes operator *A* to the first equation in (1), we have

$$\begin{array}{c} \left|\right|u_{t}{\left|\right|}_{-1}=\underset{v\in V,v\neq 0}{\sup}\frac{\left(u_{t},v\right)}{\left||v|\right|}\leq \frac{\left|(f,v)-a(u,v)-b(u,u,v)\right|}{\left||v|\right|}, \end{array}$$

which, combining with (12) and (13), implies

(21) $$\begin{array}{c} \tau \left(t\right)||u_{t}{\left(t\right)||}_{-1}^{2}+e^{-2\delta_{0}t}\int_{0}^{t}e^{2\delta_{0}\tilde{t}}\left|\right|u_{\tilde{t}}{\left|\right|}_{-1}^{2}d\tilde{t}\leq \kappa . \end{array}$$

From (10) and the LBB condition, there holds

(22) $$\begin{array}{c} \left|p\right|\leq \beta_{0}^{-1}\underset{v\in X,v\neq 0}{\sup}\frac{d(v,p)}{\left||v|\right|}\leq c(||u_{t}{\left|\right|}_{-1}+\nu ||u||+c_{1}\left|u|\;||u|\right|+\lambda_{1}^{-1/2}|f|), \end{array}$$

which implies

(23) $$\begin{array}{c} {\tau \left(t\right)|p\left(t\right)|}^{2}+e^{-2\delta_{0}t}\int_{0}^{t}e^{2\delta_{0}\tilde{t}}{\left|p\right|}^{2}d\tilde{t}\leq \kappa . \end{array}$$

Then, (17) follows from (21) and (23).

Using a similar process as above, we can obtain (18), which is omitted here. The proof is completed. □

We will use the Gronwall lemma.

**Lemma** **1** ([2,8])**.** *Let g,h,y be three locally integrable nonnegative functions on the time internal $\left[t_{0},+\infty \right)$ that, for all $t\geq t_{0}$, satisfy*

$$\begin{array}{c} y\left(t\right)+G\left(t\right)\leq C+\int_{t_{0}}^{t}h\left(\tilde{t}\right)d\tilde{t}+\int_{t_{0}}^{t}g\left(\tilde{t}\right)y\left(\tilde{t}\right)d\tilde{t}, \end{array}$$

*where G(t) is a nonnegative function on [0,+∞),andC≥0 is constant. Then,*

$$\begin{array}{c} y\left(t\right)+G\left(t\right)\leq \left(C+\int_{t_{0}}^{t}h\left(\tilde{t}\right)d\tilde{t}\right)\exp\left(\int_{t_{0}}^{t}g\left(\tilde{t}\right)d\tilde{t}\right). \end{array}$$

## 3. Finite Element Approximation

Suppose that $T_{h}$ is the partitioning of $\bar{\Omega }$, $h_{K}$ and $\rho_{K}$ are the diameter of the element *K* and the supremum of the diameter of a ball contained in *K*, respectively, and the mesh size $h=\max_{K\in T_{h}}h_{K}$, satisfying 0<h<1. In addition, assume that the partitioning is uniformly regular, that is, as *h* tends to zero, if there exists positive constants ϖ,σ>0 such that $\varpi h\leq h_{K}\leq \sigma \rho_{K}$ for any $K\in T_{h}$ (e.g., see Chapter 2–3 in [22] and Appendix A in [1] for more details).

We also introduce finite-dimensional subspaces $\left(X_{h},M_{h}\right)\subset \left(X,M\right)$ which are characterized by $T_{h}$. Two frequently used examples of the finite element spaces $\left(X_{h},M_{h}\right)$ are as follows [1]. Let $P_{l}\left(K\right)$ denote the space of polynomials of degree less than or equal to *l* on the element *K*.

**Example 1.** ***(Girault–Raviart element)**.*

$$\begin{array}{cc} \; & X_{h}=\left\{v_{h}\in C^{0}{\left(\Omega \right)}^{2}\cap X;v_{h}{|}_{K}\in P_{2}{\left(K\right)}^{2},\forall K\in T_{h}\right\},\\ \; & M_{h}=\left\{q_{h}\in C^{0}\left(\Omega \right)\cap M;q_{h}{|}_{K}\in P_{0}\left(K\right),\forall K\in T_{h}\right\}. \end{array}$$

**Example 2.** ***(Mini-element)**. We introduce $\hat{b}\in H_{0}^{1}\left(K\right)$, taking the value 1 at the barycenter of the element K in the partition $T_{h}$ and such that $0\leq \hat{b}\left(x\right)\leq 1$, which is called a “bubble function”. We then define the space*

$$\begin{array}{c} P_{1,h}^{b}=\left\{v_{h}\in C^{0}\left(\Omega \right);v_{h}{|}_{K}\in P_{1}\left(K\right)⊕\mathrm{span}\left\{\hat{b}\right\},\forall K\in T_{h}\right\}. \end{array}$$

*Then, we define*

$$\begin{array}{c} X_{h}={\left(P_{1,h}^{b}\right)}^{2}\cap X,\;\;\;M_{h}=\left\{q_{h}\in C^{0}\left(\Omega \right)\cap M;q_{h}{|}_{K}\in P_{1}\left(K\right),\;\;\forall K\in T_{h}\right\}. \end{array}$$

Moreover, we define the subspace $V_{h}$ of $X_{h}$ by

$$\begin{array}{c} V_{h}=\left\{v_{h}\in X_{h};d\left(v_{h},q_{h}\right)=0,\forall q_{h}\in M_{h}\right\}. \end{array}$$

Let $P_{h}:Y\to V_{h}$ be the $L^{2}$-orthogonal projection operators given by

$$\begin{array}{c} \left(P_{h}v,v_{h}\right)=\left(v,v_{h}\right)\;\;\;\forall v\in Y,v_{h}\in V_{h}, \end{array}$$

the discrete analogue $A_{h}=-P_{h}\Delta_{h}$ of the Stokes operator *A* The restriction of $A_{h}$ to $V_{h}$ is invertible [4]. Using the inverse function $A_{h}^{-1}$, which is self-adjoint and positive definite, we may define “discrete” Sobolev norms on $V_{h}$ for any order r∈R, by setting $\left|\right|v_{h}{\left|\right|}_{r}=\left|A_{h}^{r/2}v_{h}\right|\;\;\;\forall v_{h}\in V_{h}$.

(**A3**) For the finite element spaces $\left(X_{h},M_{h}\right)$, we assume that the following approximation properties hold: for all $v\in D\left(A\right),q\in H^{1}\left(\Omega \right)\cap M,$ there exist operators $I_{h}v\in X_{h}$ and $J_{h}q\in M_{h}$ such that

(24) $$\begin{array}{cc} \; & |v-I_{h}v|+h||v-I_{h}v||\leq ch^{2}{\left||v|\right|}_{2}, \end{array}$$

(25) $$\begin{array}{cc} \; & |q-J_{h}{q|\leq ch||q||}_{1}, \end{array}$$

together with the inverse inequality

(26) $$\begin{array}{c} \left|\right|v_{h}\left|\right|\leq c_{2}h^{-1}\left|v_{h}\right|\;\;\forall v_{h}\in X_{h}, \end{array}$$

and the discrete LBB condition (see, e.g., Theorem 1.1 of Chapter II in [1]): for each $q_{h}\in M_{h},$ there exists a positive constant $\beta_{0}^{*}$ and $v_{h}\in X_{h},v_{h}\neq 0$ such that

(27) $$\begin{array}{c} \underset{v_{h}\in X_{h},v_{h}\neq 0}{\sup}\frac{d(v_{h},q_{h})}{∥v_{k}∥}\geq \beta_{0}^{*}\left|q_{h}\right|. \end{array}$$

The following properties are classical (see [1,3]):

(28) $$\begin{array}{cc} \; & \left|\right|P_{h}v||\leq c||v||\;\;\forall v\in X, \end{array}$$

(29) $$\begin{array}{cc} \; & |v-P_{h}v|+h||v-P_{h}v||\leq ch^{2}{\left||v|\right|}_{2}\;\;\forall v\in D\left(A\right), \end{array}$$

(30) $$\begin{array}{cc} \; & |v-P_{h}v|\leq ch||v-P_{h}v{\left|\right|}_{1}\;\;\forall v\in X. \end{array}$$

With the above notations, the finite element semi-discrete approximation of the problems (10) and (11) reads: Find $\left(u_{h},p_{h}\right)\in \left(X_{h},M_{h}\right)$ such that for all $t>0,\left(v,q\right)\in \left(X_{h},M_{h}\right),$

(31) $$\begin{array}{cc} \; & \left(u_{ht},v\right)+a\left(u_{h},v\right)-d\left(v,p_{h}\right)+d\left(u_{h},q\right)+b\left(u_{h},u_{h},v\right)=\left(f,v\right), \end{array}$$

(32) $$\begin{array}{cc} \; & u_{h}\left(0\right)=u_{0h}=P_{h}u_{0}. \end{array}$$

For the finite element approximation problems (31) and (32), applying the same manner as that in Theorem 1 above and Proposition 3.2 of [4], we obtain that

**Theorem** **2.** *Under the assumptions of (* ***A1*** *)–(* ***A3*** *), the solution of the problems (31) and (32) satisfies the following bounds for all time t>0*

(33) $$\begin{array}{c} |u_{h}{\left(t\right)|}^{2}+e^{-2\delta_{0}t}\int_{0}^{t}e^{2\delta_{0}\tilde{t}}\left(|\right|u_{h}{\left|\right|}^{2}+|p_{h}{|}^{2})d\tilde{t}\leq \kappa , \end{array}$$

(34) $$\begin{array}{c} \lim_{t\to \infty }\sup||u_{h}\left(t\right)||\leq \nu^{-1}\left|\right|f_{\infty }{\left|\right|}_{-1}, \end{array}$$

(35) $$\begin{array}{c} \tau \left(t\right)(||u_{h}{\left(t\right)||}^{2}+|p_{h}{\left(t\right)|}^{2})+e^{-2\delta_{0}t}\int_{0}^{t}e^{2\delta_{0}\tilde{t}}\tau \left(\tilde{t}\right)\left(\right|A_{h}u_{h}{|}^{2}+|u_{h\tilde{t}}{|}^{2}+\left|\right|p_{h}{\left|\right|}_{1}^{2})d\tilde{t}\leq \kappa ,\\ \tau^{2}\left(t\right)(|A_{h}u_{h}{\left(t\right)|}^{2}+|u_{ht}{\left(t\right)|}^{2}+\left|\right|p_{h}{\left(t\right)||}_{1}^{2})\;\;\;\;\;\;\;\;\;\;\;\; \end{array}$$

(36) $$\begin{array}{c} +e^{-2\delta_{0}t}\int_{0}^{t}e^{2\delta_{0}\tilde{t}}\tau^{2}\left(\tilde{t}\right)\left(|\right|u_{h\tilde{t}}{\left|\right|}^{2}+|p_{h\tilde{t}}{|}^{2})d\tilde{t}\leq \kappa , \end{array}$$

(37) $$\begin{array}{c} \tau^{3}\left(t\right)||u_{ht}{\left(t\right)||}^{2}+e^{-2\delta_{0}t}\int_{0}^{t}e^{2\delta_{0}\tilde{t}}\tau^{3}\left(\tilde{t}\right)\left(\right|A_{h}u_{h\tilde{t}}{|}^{2}+|u_{h\tilde{t}\tilde{t}}{|}^{2}+\left|\right|p_{h\tilde{t}}{\left|\right|}_{1}^{2})d\tilde{t}\leq \kappa . \end{array}$$

## 4. Uniform Error Estimates

In this section, we discuss error estimates for the finite element approximation. Because of the singularity of the solution on t∈[0,1), we first need to introduce an intermediate step which is defined by a finite element Galerkin approximation to the linearized Navier–Stokes equations: Find $\left(\tilde{u},\tilde{p}\right)\in \left(X_{h},M_{h}\right)$ such that for all t>0

(38) $$\begin{array}{cc} \; & \left({\tilde{u}}_{ht},v\right)+a\left({\tilde{u}}_{h},v\right)+d\left(v,{\tilde{p}}_{h}\right)-d\left({\tilde{u}}_{h},q\right)=\left(f,v\right)-b\left(u,u,v\right),\;\;\;\forall \left(v,q\right)\in \left(X_{h},M_{h}\right) \end{array}$$

(39) $$\begin{array}{cc} \; & {\tilde{u}}_{0h}=P_{h}u_{0}. \end{array}$$

Setting the finite approximation error $e_{h}=u-u_{h}$, it follows that

(40) $$\begin{array}{c} e_{h}=u-{\tilde{u}}_{h}+{\tilde{u}}_{h}-u_{h}:=\xi_{h}+\eta_{h}, \end{array}$$

where $\xi_{h}$ is the error generated by the finite element approximation of the linearized system (38) and (39), and $\eta_{h}$ represents the error coming from the nonlinear term.

To give the optimal error estimate for $\xi_{h}$, we also need to recall the Stokes projection. For u∈V and p∈M, define $S_{h}u\in V_{h}$ by

(41) $$\begin{array}{c} a\left(u-S_{h}u,v\right)=\left(p,\nabla \cdot v\right),\;\;\;\forall v\in V_{h}, \end{array}$$

with $S_{h}u_{0}=P_{h}u_{0}$.

We have the following lemma for the Stokes projection:

**Lemma** **2.** *Supposing $S_{h}u$ is defined by (41), then, there hold, for k=1,2,*

(42) $$\begin{array}{cc} |u-S_{h}{u|}^{2}+h^{2}||u-S_{h}u{\left|\right|}^{2}\leq & ch^{2k}{\left(||u|\right|}_{k}^{2}+{\left||p|\right|}_{k-1}^{2}), \end{array}$$

(43) $$\begin{array}{cc} |u_{t}-S_{h}u_{t}{|}^{2}+h^{2}\left|\right|u_{t}-S_{h}u_{t}{\left|\right|}^{2}\leq & ch^{2k}\left(\right|u_{t}{|}_{k}^{2}+\left|\right|p_{t}{\left|\right|}_{k-1}^{2}), \end{array}$$

(44) $$\begin{array}{cc} ||u-S_{h}u{\left|\right|}_{-1}^{2}\leq & ch^{2(k+1)}{\left(||u|\right|}_{k}^{2}+{\left||p|\right|}_{k}^{2}), \end{array}$$

(45) $$\begin{array}{cc} \left|\right|u_{t}-S_{h}u_{t}{\left|\right|}_{-1}^{2}\leq & ch^{2(k+1)}\left(|\right|u_{t}{\left|\right|}_{k}^{2}+\left|\right|p_{t}{\left|\right|}_{k}^{2}). \end{array}$$

**Proof.**  The results in (42) and (43) are classical, which can be found in [3]. To derive (44) and (45), we consider the following dual problem: Find (w,z)∈(X,M) such that

(46) $$\begin{array}{cc} -\Delta w+\nabla z & =u-S_{h}u,\;\;\;\;\mathrm{in}\;\Omega , \end{array}$$

(47) $$\begin{array}{cc} \nabla \cdot w & =0, \end{array}$$

which follows that, after simple calculation,

(48) $$\begin{array}{c} {\left||w|\right|}_{2}+{\left||z|\right|}_{1}\leq c\left|u-S_{h}u\right|. \end{array}$$

Therefore, it holds that

$$\begin{array}{cc} ||u-S_{h}u{\left|\right|}_{-1}^{2} & =(w,u-S_{h}u)\\ \; & =(w-I_{h}w,u-S_{h}u)\\ \; & \leq c||u-S_{h}{u||}_{-1}||w-I_{h}w{\left|\right|}_{1}. \end{array}$$

That is,

$$\begin{array}{c} ||u-S_{h}{u||}_{-1}\leq {ch||w||}_{2}\leq ch||u-S_{h}u||\leq ch^{k+1}{\left(||u|\right|}_{k+1}+{\left||p|\right|}_{k}), \end{array}$$

which implies (44). Applying a similar process, we can obtain the inequality (45). This completes the proof. □

In line with the notation introduced in (40), we thus find

$$\begin{array}{c} \xi_{h}=u-{\tilde{u}}_{h}=u-S_{h}u+S_{h}u-{\tilde{u}}_{h}:=w_{h}+\theta_{h}, \end{array}$$

with $\theta_{h}\left(0\right)=0$, which means that we split $\xi_{h}$ into two parts, $w_{h}$ and $\theta_{h}$. Since bounds for the first part $w_{h}$ were given in Lemma 2, we only need to analyze the second part $\theta_{h}$ in the following. Then, the estimates for $\xi_{h}$ follow directly.

Due to (10), (38) and (41), we have

(49) $$\begin{array}{c} \left(\theta_{ht},v\right)+a\left(\theta_{h},v\right)=-\left(w_{ht},v\right),\;\;\;\forall v\in V_{h}. \end{array}$$

Note that for estimates of $|w_{ht}|$ in (49), the introduction of the τ(t) term is necessary so that we can avoid nonlocal compatibility conditions [2,3,4]. Firstly, we introduce the following symbol:

$$\begin{array}{c} {\hat{\theta }}_{h}\left(t\right)=\int_{0}^{t}\theta_{h}\left(\tilde{t}\right)d\tilde{t}. \end{array}$$

The above equation implies that

$$\begin{array}{c} \frac{d}{dt}{\hat{\theta }}_{h}\left(t\right)=\theta_{h}\left(t\right)\;\mathrm{and}\;{\hat{\theta }}_{h}\left(0\right)=0. \end{array}$$

Integrating (49) from 0 to *t* and noting that $\left(u_{0}-P_{h}u_{0},v\right)=0,\forall v\in V_{h}$, we obtain

(50) $$\begin{array}{c} \left(\theta_{h},v\right)+a\left({\hat{\theta }}_{h},v\right)=-\left(w_{h},v\right),\;\;\;\forall v\in V_{h}. \end{array}$$

**Lemma** **3.** *Under the assumptions of Theorem 2, we have, for all t>0,*

(51) $$\begin{array}{cc} |{\hat{\theta }}_{h}{\left(t\right)|}^{2}+e^{-2\delta_{0}t}\int_{0}^{t}e^{2\delta_{0}\tilde{t}}\left(|\right|{\hat{\theta }}_{h}{\left|\right|}^{2}+\left|\right|\theta_{h}{\left|\right|}_{-1}^{2})d\tilde{t}\leq & ch^{4}, \end{array}$$

(52) $$\begin{array}{cc} \tau \left(t\right)(||{\hat{\theta }}_{h}{\left(t\right)||}^{2}+\left|\right|\theta_{h}{\left(t\right)||}_{-1}^{2})+e^{-2\delta_{0}t}\int_{0}^{t}e^{2\delta_{0}\tilde{t}}\tau \left(\tilde{t}\right){\left|\theta_{h}\right|}^{2}d\tilde{t}\leq & ch^{4}, \end{array}$$

(53) $$\begin{array}{cc} \tau^{2}\left(t\right)|\theta_{h}{\left(t\right)|}^{2}+e^{-2\delta_{0}t}\int_{0}^{t}e^{2\delta_{0}\tilde{t}}\tau^{2}\left(\tilde{t}\right)\left|\right|\theta_{h}{\left|\right|}^{2}d\tilde{t}\leq & ch^{4}, \end{array}$$

(54) $$\begin{array}{cc} \tau^{3}\left(t\right)||\theta_{h}{\left(t\right)||}^{2}+e^{-2\delta_{0}t}\int_{0}^{t}e^{2\delta_{0}\tilde{t}}\tau^{3}\left(\tilde{t}\right){\left|\theta_{h\tilde{t}}\right|}^{2}d\tilde{t}\leq & ch^{4}. \end{array}$$

**Proof.**  Setting $v=e^{2\delta_{0}t}{\hat{\theta }}_{h}$ in (50) and noting that

$$\begin{array}{c} \frac{\nu }{2}\left|\right|{\hat{\theta }}_{h}{\left|\right|}^{2}\geq \frac{\nu \lambda_{1}}{2}|{\hat{\theta }}_{h}{|}^{2}\geq \delta_{0}{\left|{\hat{\theta }}_{h}\right|}^{2}, \end{array}$$

we obtain

(55) $$\begin{array}{c} \frac{1}{2}\frac{d}{dt}(e^{2\delta_{0}t}|{\hat{\theta }}_{h}{|}^{2})+\frac{\nu }{2}e^{2\delta_{0}t}\left|\right|{\hat{\theta }}_{h}{\left|\right|}^{2}\leq -e^{2\delta_{0}t}\left(w_{h},{\hat{\theta }}_{h}\right). \end{array}$$

By the Young inequality and (3), we have

$$\begin{array}{c} |-\left(w_{h},{\hat{\theta }}_{h}\right)|\leq \frac{\nu }{4}\left|\right|{\hat{\theta }}_{h}{\left|\right|}^{2}+\frac{1}{\nu }\left|\right|w_{h}{\left|\right|}_{-1}^{2}. \end{array}$$

Taking this estimate into (55), then integrating it from 0 to *t* and using (44) with k=1, (12) and (17), one finds

(56) $$\begin{array}{cc} |{\hat{\theta }}_{h}{\left(t\right)|}^{2}+e^{-2\delta_{0}t}\int_{0}^{t}e^{2\delta_{0}\tilde{t}}\left|\right|{\hat{\theta }}_{h}{\left|\right|}^{2}d\tilde{t}\leq & ch^{4}, \end{array}$$

after a final multiplying by $e^{-2\delta_{0}t}$. Taking $v=e^{2\delta_{0}t}A_{h}^{-1}\theta_{h}$ in (50) yields

$$\begin{array}{c} e^{2\delta_{0}t}{\left||\theta |\right|}_{-1}^{2}+\frac{\nu }{2}\frac{d}{dt}e^{2\delta_{0}t}|\hat{\theta }{|}^{2}=\delta_{0}\nu {\left|\hat{\theta }\right|}^{2}-e^{2\delta_{0}t}\left(w_{h},A^{-1}\theta_{h}\right). \end{array}$$

Applying

$$\begin{array}{c} |-e^{2\delta_{0}t}\left(w_{h},A^{-1}\theta_{h}\right)|\leq \frac{1}{2}e^{2\delta_{0}t}\left|\right|w_{h}{\left|\right|}_{-1}^{2}+\frac{1}{2}e^{2\delta_{0}t}{\left||\theta |\right|}_{-1}^{2} \end{array}$$

in the above equation, using (44) with k=1 and Theorem 1, then multiplying the resulting inequality by $e^{-2\delta_{0}t}$ and considering (56), we obtain (51).

Choosing $v=e^{2\delta_{0}t}\theta_{h}$ in (50), we find

(57) $$\begin{array}{c} e^{2\delta_{0}t}\tau \left(t\right)|\theta_{h}{|}^{2}+\frac{\nu }{2}\frac{d}{dt}e^{2\delta_{0}t}\tau \left(t\right)||{\hat{\theta }}_{h}{\left|\right|}^{2}\leq -e^{2\delta_{0}t}\tau \left(t\right)\left(w_{h},\theta_{h}\right)+\left(\frac{\nu }{2}+\nu \delta_{0}\right)e^{2\delta_{0}t}\left|\right|{\hat{\theta }}_{h}{\left|\right|}^{2}. \end{array}$$

There holds

$$\begin{array}{cc} |-(w_{h},\theta_{h})|\leq & \frac{1}{2}|\theta_{h}{|}^{2}+\frac{1}{2}{\left|w_{h}\right|}^{2}. \end{array}$$

Combining this inequality with (57), integrating it concerning the time from 0 to *t*, then multiplying it by $e^{-2\delta_{0}t}$ and using (42), (13), (18) and (51), we obtain

(58) $$\begin{array}{c} \tau \left(t\right)||{\hat{\theta }}_{h}{\left(t\right)||}^{2}+e^{-2\delta_{0}t}\int_{0}^{t}e^{2\delta_{0}\tilde{t}}\tau \left(\tilde{t}\right){\left|\theta_{h}\right|}^{2}d\tilde{t}\leq ch^{4}. \end{array}$$

Setting $v=e^{2\delta_{0}t}A_{h}^{-1}\theta_{h}$ in (49), it holds that

$$\begin{array}{c} \frac{1}{2}\frac{d}{dt}e^{2\delta_{0}t}\tau \left(t\right)|\theta_{h}{|}^{2}+\nu e^{2\delta_{0}t}\tau \left(t\right)|\theta_{h}{|}^{2}=-e^{2\delta_{0}t}\tau \left(t\right)\left(w_{ht},A^{-1}\theta_{h}\right)+\left(\frac{1}{2}+\delta_{0}\right)e^{2\delta_{0}t}\left|\right|{\hat{\theta }}_{h}{\left|\right|}^{2}. \end{array}$$

Applying

$$\begin{array}{c} |-e^{2\delta_{0}t}\tau \left(t\right)\left(w_{ht},A_{h}^{-1}\theta_{h}\right)|\leq -\frac{1}{2}e^{2\delta_{0}t}\tau \left(t\right)\left(|\right|w_{ht}{\left|\right|}_{-1}^{2}+\left|\right|\theta_{h}{\left|\right|}_{-1}^{2}), \end{array}$$

in the above equation, using (45) and Theorem 1, we have (52) by considering (58).

Moreover, taking $v=e^{2\delta_{0}t}\tau \left(t\right)\theta_{h}$ in (49) and noting that $\frac{d}{dt}\tau^{2}\left(t\right)\leq 2\tau \left(t\right)$, one finds that

(59) $$\begin{array}{cc} \frac{1}{2}\frac{d}{dt}(e^{2\delta_{0}t}\tau^{2}\left(t\right)|\theta_{h}{|}^{2})+ & \nu e^{2\delta_{0}t}\tau^{2}\left(t\right)||\theta_{h}{\left|\right|}^{2}\leq -e^{2\delta_{0}t}\left(w_{ht},\tau^{2}\left(t\right)\theta_{h}\right)+\left(\frac{\nu }{2}+\delta_{0}\right)e^{2\delta_{0}t}\tau \left(t\right){\left|\theta_{h}\right|}^{2}. \end{array}$$

Since

$$\begin{array}{c} |-\left(w_{ht},\tau^{2}\left(t\right)\theta_{h}\right)|\leq \tau^{2}\left(t\right)|w_{ht}\left|\;\right|\theta_{h}|\leq \tau^{3}\left(t\right)|w_{ht}{|}^{2}+\frac{1}{4}\tau \left(t\right){\left|\theta_{h}\right|}^{2}, \end{array}$$

integrating (59) from 0 to *t*, using (52), (43), (15) and (18), we have (53) by a final multiplying by $e^{-2\delta_{0}t}$.

Finally, setting $v=e^{2\delta_{0}t}\tau^{3}\left(t\right)\theta_{ht}$ in (49), it follows that

(60) $$\begin{array}{cc} \; & e^{2\delta_{0}t}\tau^{3}\left(t\right)|\theta_{ht}{|}^{2}+\frac{\nu }{2}\frac{d}{dt}(e^{2\delta_{0}t}\tau^{3}\left(t\right)||\theta_{h}{\left|\right|}^{2})\\ \leq & -\left(w_{ht},e^{2\delta_{0}t}\tau^{3}\left(t\right)\theta_{ht}\right)+\nu \left(\delta_{0}+1\right)e^{2\delta_{0}t}\tau^{2}\left(t\right)\left|\right|\theta_{h}{\left|\right|}^{2}. \end{array}$$

Due to

$$\begin{array}{c} |\left(w_{ht},\tau^{3}\left(t\right)\theta_{ht}\right)|\leq \frac{1}{2}\tau^{3}\left(t\right)|\theta_{ht}{|}^{2}+\frac{1}{2}\tau^{3}\left(t\right){\left|w_{ht}\right|}^{2}, \end{array}$$

introducing this inequality into (60), integrating the resulting inequality from 0 to *t* then multiplying by $e^{-2\delta_{0}t}$, we deduce that

$$\begin{array}{cc} \; & \nu \tau^{3}\left(t\right)||\theta_{h}{\left|\right|}^{2}+e^{-2\delta_{0}t}\int_{0}^{t}e^{2\delta_{0}\tilde{t}}\tau^{3}\left(\tilde{t}\right){\left|\theta_{h\tilde{t}}\right|}^{2}d\tilde{t}\\ \leq & 2e^{-2\delta_{0}t}\int_{0}^{t}e^{2\delta_{0}\tilde{t}}\nu \left(\delta_{0}+1\right)\tau^{2}\left(\tilde{t}\right)\left|\right|\theta_{h}{\left|\right|}^{2}d\tilde{t}+e^{-2\delta_{0}t}\int_{0}^{t}e^{2\delta_{0}\tilde{t}}\tau^{3}\left(\tilde{t}\right){\left|w_{h\tilde{t}}\right|}^{2}d\tilde{t}. \end{array}$$

using (53), (43), (15) and (18), then the proof is completed. □

Theorem 2 and Lemmas 2 and 3 imply the following.

**Lemma** **4.** *Under the assumptions of Theorem 2, we have, for all t>0, that*

(61) $$\begin{array}{cc} e^{-2\delta_{0}t}\int_{0}^{t}e^{2\delta_{0}\tilde{t}}\left|\right|\xi_{h}{\left|\right|}_{-1}^{2}d\tilde{t}\leq & ch^{4}, \end{array}$$

(62) $$\begin{array}{cc} \tau \left(t\right)||\xi_{h}{\left(t\right)||}_{-1}^{2}+e^{-2\delta_{0}t}\int_{0}^{t}e^{2\delta_{0}\tilde{t}}\tau \left(\tilde{t}\right){\left|\xi_{h}\right|}^{2}d\tilde{t}\leq & ch^{4}, \end{array}$$

(63) $$\begin{array}{cc} \tau^{2}\left(t\right)|\xi_{h}{\left(t\right)|}^{2}+e^{-2\delta_{0}t}\int_{0}^{t}e^{2\delta_{0}\tilde{t}}\tau^{2}\left(\tilde{t}\right)\left|\right|\xi_{h}{\left|\right|}^{2}d\tilde{t}\leq & ch^{4}, \end{array}$$

(64) $$\begin{array}{cc} \tau^{3}\left(t\right)||\xi_{h}{\left(t\right)||}^{2}+e^{-2\delta_{0}t}\int_{0}^{t}e^{2\delta_{0}\tilde{t}}\tau^{3}\left(\tilde{t}\right){\left|\xi_{h\tilde{t}}\right|}^{2}d\tilde{t}\leq & ch^{4}. \end{array}$$

Lemma 4 provides the error bounds generated by the finite element approximation to the linearized Navier–Stokes when the initial data belong to the $L^{2}\left(\Omega \right)$ space. Next, we consider the errors from the nonlinear terms. The long-term behavior of the finite element error is discussed below.

**Lemma** **5.** *Under the assumptions of Theorem 2, if*

(65) $$\begin{array}{c} N\nu^{-2}\left|\right|f_{\infty }{\left|\right|}_{-1}<1, \end{array}$$

*there holds*

(66) $$\begin{array}{c} \lim_{t\to \infty }\sup||\eta_{h}\left(t\right)\left|\right|\leq ch. \end{array}$$

**Proof.**  Subtracting (31) from (38), we arrive at

(67) $$\begin{array}{c} \left(\eta_{ht},v\right)+a\left(\eta_{h},v\right)+b\left(e_{h},u_{h},v\right)+b\left(u,e_{h},v\right)=0, \end{array}$$

with $\eta_{h}\left(0\right)=0.$

Taking $v=e^{2\delta_{0}t}\eta_{h}$ in (67) and using (5), we have

(68) $$\begin{array}{c} \frac{1}{2}\frac{d}{dt}(e^{2\delta_{0}t}|\eta_{h}{|}^{2})+\nu e^{2\delta_{0}t}\left|\right|\eta_{h}{\left|\right|}^{2}=\delta_{0}e^{2\delta_{0}t}{\left|\eta_{h}\right|}^{2}-e^{2\delta_{0}t}\left[b\left(e_{h},u_{h},\eta_{h}\right)+b\left(u,\xi_{h},\eta_{h}\right)\right]. \end{array}$$

By (10), it holds true that

$$\begin{array}{cc} \left|b(e_{h},u_{,}\eta_{h})\right| & \leq N||e_{h}\left||\;|\right|u_{h}\left||\;|\right|\eta_{h}\left|\right|\leq N(||\xi_{h}\left|\right|+\left|\right|\eta_{h}\left||)|\right|u_{h}\left||\;|\right|\eta_{h}\left|\right|,\\ \left|b(u,\xi_{h},\eta_{h})\right| & \leq N||u||\;||\xi_{h}\left||\;|\right|\eta_{h}\left|\right|. \end{array}$$

Combining these estimates with (78), integrating it from 0 to *t*, and multiplying by $e^{-2\delta_{0}t}$, one finds that

(69) $$\begin{array}{cc} \; & |\eta_{h}{\left(t\right)|}^{2}+2e^{-2\delta_{0}t}\int_{0}^{t}e^{2\delta_{0}\tilde{t}}\left(\nu -N|\right|u_{h}\left||)|\right|\eta_{h}{\left|\right|}^{2}d\tilde{t}\\ \leq & 2\delta_{0}e^{-2\delta_{0}t}\int_{0}^{t}e^{2\delta_{0}\tilde{t}}|\eta_{h}{|}^{2}d\tilde{t}+2Ne^{-2\delta_{0}t}\int_{0}^{t}e^{2\delta_{0}\tilde{t}}\left(||u|\right|+\left|\right|u_{h}\left||)|\right|\eta_{h}\left||\;|\right|\xi_{h}\left|\right|d\tilde{t}. \end{array}$$

Letting t→∞ and using the L’Hospital rule, it follows that

$$\begin{array}{cc} \lim_{t\to \infty }2e^{-2\delta_{0}t}\int_{0}^{t}e^{2\delta_{0}\tilde{t}}\left(\nu -N|\right|u_{h}\left||)|\right|\eta_{h}{\left|\right|}^{2}d\tilde{t}= & 2\lim_{t\to \infty }\frac{\int_{0}^{t}e^{2\delta_{0}\tilde{t}}\left(\nu -N|\right|u_{h}\left||)|\right|\eta_{h}{\left|\right|}^{2}d\tilde{t}}{e^{2\delta_{0}t}}\\ = & 2\lim_{t\to \infty }\frac{e^{2\delta_{0}t}\left(\nu -N|\right|u_{h}\left(t\right)||)||\eta_{h}\left(t\right){\left|\right|}^{2}}{2\delta_{0}e^{2\delta_{0}t}}\\ = & \delta_{0}^{-1}(\nu -N\lim_{t\to \infty }\left|\right|u_{h}\left(t\right)||)\lim_{t\to \infty }\left|\right|\eta_{h}\left(t\right){\left|\right|}^{2},\\ \lim_{t\to \infty }2\delta_{0}e^{-2\delta_{0}t}\int_{0}^{t}e^{2\delta_{0}\tilde{t}}{\left|\eta_{h}\right|}^{2}d\tilde{t}= & \lim_{t\to \infty }2\delta_{0}\frac{\int_{0}^{t}e^{2\delta_{0}\tilde{t}}{\left|\eta_{h}\right|}^{2}d\tilde{t}}{e^{2\delta_{0}t}}=\lim_{t\to \infty }{\left|\eta_{h}\left(t\right)\right|}^{2},\\ \lim_{t\to \infty }2Ne^{-2\delta_{0}t}\int_{0}^{t}e^{2\delta_{0}\tilde{t}}\left(||u|\right|+\left|\right|u_{h}\left||)|\right|\eta_{h}\left||\;|\right|\xi_{h}\left|\right|d\tilde{t}= & \delta_{0}^{-1}N\lim_{t\to \infty }\left(||u\left(t\right)|\right|+\left|\right|u_{h}\left(t\right)||)||\eta_{h}\left(t\right)||\;||\xi_{h}\left(t\right)\left|\right|. \end{array}$$

Inputting the above equations into (69), taking the limitation concerning the time, using Theorems 1 and 2 and Lemma 4, and noting that $\lim_{t\to \infty }\tau \left(t\right)=1$, we obtain

(70) $$\begin{array}{c} \lim_{t\to \infty }\sup||\eta_{h}\left(t\right){\left|\right|}^{2}\leq ch^{2}. \end{array}$$

The proof is completed. □

**Lemma** **6.** *Under the assumption of Lemma 5, we have, for all t≥0,*

(71) $$\begin{array}{cc} |{\hat{\eta }}_{h}{|}^{2}+e^{-2\delta_{0}t}\int_{0}^{t}e^{2\delta_{0}\tilde{t}}\left|\right|\eta_{h}\left(\tilde{t}\right){\left|\right|}_{-1}^{2}d\tilde{t}\leq & ch^{2}, \end{array}$$

(72) $$\begin{array}{cc} \tau \left(t\right)||{\hat{\eta }}_{h}{\left|\right|}^{2}+e^{-2\delta_{0}t}\int_{0}^{t}e^{2\delta_{0}\tilde{t}}\tau \left(\tilde{t}\right){\left|\eta_{h}\left(\tilde{t}\right)\right|}^{2}d\tilde{t}\leq & ch^{2}. \end{array}$$

**Proof.**  We first consider the case when t∈[0,1]. Integrating (67) from 0 to *t* and noting that

$$\int_{0}^{t}\left(e_{h}\cdot \nabla \right)u_{h}d\tilde{t}=\int_{0}^{t}\nabla u_{h}d{\hat{e}}_{h}=\left({\hat{e}}_{h}\cdot \nabla \right)u_{h}{|}_{0}^{t}-\int_{0}^{t}\left({\hat{e}}_{h}\cdot \nabla \right)u_{h\tilde{t}}d\tilde{t},$$

we obtain

(73) $$\begin{array}{cc} \left(\eta_{h},v\right)+a\left({\hat{\eta }}_{h},v\right) & +b\left({\hat{e}}_{h},u_{h},v\right)-b\left({\hat{e}}_{h}\left(0\right),u_{h}\left(0\right),v\right)+b\left(u,{\hat{e}}_{h},v\right)-b\left(u_{h}\left(0\right),{\hat{e}}_{h}\left(0\right),v\right)\\ \; & +\left(\int_{0}^{t}\left[B\left({\hat{e}}_{h},u_{h\tilde{t}}\right)+B\left(u_{\tilde{t}},{\hat{e}}_{h}\right)\right]d\tilde{t},v\right)=0. \end{array}$$

Taking $v=A_{h}^{-1}\eta_{h}$ and using

$$\begin{array}{cc} \; & \left|-\left(\int_{0}^{t}\left[B\left({\hat{e}}_{h},u_{h\tilde{t}}\right)+B\left(u_{\tilde{t}},{\hat{e}}_{h}\right)\right]d\tilde{t},A_{h}^{-1}\eta_{h}\right)\right|\\ \leq & c\tau \left(t\right)|{\hat{e}}_{h}{|}^{1/2}\left|\right|{\hat{e}}_{h}{\left|\right|}^{1/2}\left(\right|u_{ht}{|}^{1/2}\left|\right|u_{ht}{\left|\right|}^{1/2}+|u_{t}{\left|\right|}^{1/2}\left|\right|u_{t}{\left|\right|}^{1/2})||\eta_{h}{\left|\right|}_{-1},\\ |b({\hat{e}}_{h},u_{h},v)|\leq & c||u_{h}{\left|\right|}^{1/2}|A_{h}u_{h}{|}^{1/2}|{\hat{e}}_{h}\left|\;|\right|{\hat{e}}_{h}{\left|\right|}_{-1}, \end{array}$$

in (73), then integrating from 0 to *t* and using Lemmas 1 and 4–6, and Theorems 1 and 2, we deduce that

(74) $$\begin{array}{cc} \; & |{\hat{\eta }}_{h}{\left(t\right)|}^{2}+\int_{0}^{t}\left|\right|\eta_{h}{\left|\right|}_{-1}^{2}d\tilde{t}\leq ch^{2}. \end{array}$$

Moreover, taking $v={\hat{\eta }}_{h}$ and $v=\tau \left(t\right)\eta_{h}$ in (73), respectively, and following a similar process, we have

(75) $$\begin{array}{cc} \; & \tau \left(t\right)||{\hat{\eta }}_{h}{\left(t\right)||}^{2}+\int_{0}^{t}\tau \left(\tilde{t}\right){\left|\eta_{h}\right|}^{2}d\tilde{t}\leq ch^{2}. \end{array}$$

When t∈(1,+∞), it is easily derived by according the classical process. The proof is completed. □

**Lemma** **7.** *Under the assumptions of Lemma 5, we have, for all t>0,*

(76) $$\begin{array}{cc} \tau^{2}\left(t\right)|\eta_{h}{\left(t\right)|}^{2}+e^{-2\delta_{0}t}\int_{0}^{t}e^{2\delta_{0}\tilde{t}}\tau^{2}\left(\tilde{t}\right)\left|\right|\eta_{h}{\left|\right|}^{2}d\tilde{t}\leq & ch^{2}, \end{array}$$

(77) $$\begin{array}{cc} \tau^{2}\left(t\right)||\eta_{h}{\left(t\right)||}^{2}+e^{-2\delta_{0}t}\int_{0}^{t}e^{2\delta_{0}\tilde{t}}\tau^{2}\left(\tilde{t}\right){\left|\eta_{h\tilde{t}}\right|}^{2}d\tilde{t}\leq & ch^{2}. \end{array}$$

**Proof.**  Since there exists a sufficiently large enough *T* such that [0,+∞)=(0,T]∪(T,+∞) with (T,+∞) being the neighborhood of +∞ in which the inequality (66) holds, first, we consider the error on the domain t∈(0,T]. Taking $v=\tau^{2}\left(t\right)\eta_{h}$ in (67), it follows that

(78) $$\begin{array}{c} \frac{1}{2}\frac{d}{dt}(\tau^{2}\left(t\right)|\eta_{h}{|}^{2})+\nu \tau^{2}\left(t\right)||\eta_{h}{\left|\right|}^{2}=\tau \left(t\right){\left|\eta_{h}\right|}^{2}-\tau^{2}\left(t\right)\left[b\left(e_{h},u_{h},\eta_{h}\right)+b\left(u,\xi_{h},\eta_{h}\right)\right]. \end{array}$$

Using (5) and (6), there hold that

$$\begin{array}{cc} \tau^{2}\left(t\right)\left|b\left(\xi_{h},u_{h},\eta_{h}\right)\right|\leq & \tau^{2}\left(t\right)c_{1}\left(\right|\xi_{h}{|}^{1/2}\left|\right|\xi_{h}{\left|\right|}^{1/2}\left|\right|u_{h}\left|\right|+\left|\right|\xi_{h}\left||\;\right|u_{h}{|}^{1/2}\left|\right|u_{h}{\left|\right|}^{1/2})|\eta_{h}{|}^{1/2}\left|\right|\eta_{h}{\left|\right|}^{1/2}\\ \leq & \tau^{2}\left(t\right)\left(c_{1}^{2}\left|\right|u_{h}{\left|\right|}^{2}|\xi_{h}\left|\;|\right|\xi_{h}\left|\right|+\frac{1}{4}|\eta_{h}\left|\;|\right|\eta_{h}\left|\right|+c_{1}^{2}\left|\right|u_{h}\left||\;|\right|\xi_{h}{\left|\right|}^{2}+\frac{1}{4}|u_{h}\left|\;\right|\eta_{h}\left|\;|\right|\eta_{h}\left|\right|\right)\\ \leq & \tau^{2}\left(t\right)(c_{1}^{2}\left|\right|u_{h}{\left|\right|}^{2}|\xi_{h}\left|\;|\right|\xi_{h}\left|\right|+c_{1}^{2}\left|\right|u_{h}\left||\;|\right|\xi_{h}{\left|\right|}^{2}\\ \; & +\frac{\nu }{8}\left|\right|\eta_{h}{\left|\right|}^{2}+\frac{1}{\nu }\left(\right|\eta_{h}{|}^{2}+|u_{h}{|}^{2}|\eta_{h}{|}^{2}))\\ \tau^{2}\left(t\right)\left|b\left(\eta_{h},u_{h},\eta_{h}\right)\right|\leq & \tau^{2}\left(t\right)c_{1}\left(\right|\eta_{h}{|}^{1/2}\left|\right|\eta_{h}{\left|\right|}^{1/2}\left|\right|u_{h}\left|\right|+\left|\right|\eta_{h}\left||\;\right|u_{h}{|}^{1/2}\left|\right|u_{h}{\left|\right|}^{1/2})|\eta_{h}{|}^{1/2}\left|\right|\eta_{h}{\left|\right|}^{1/2}\\ \leq & \frac{\nu }{16}\tau^{2}\left(t\right)||\eta_{h}{\left|\right|}^{2}+\frac{2}{\nu }c_{1}^{2}\tau^{2}\left(t\right)||u_{h}{\left|\right|}^{2}|\eta_{h}{|}^{2}+{\left(\frac{4}{\nu }\right)}^{4}c_{1}^{4}\tau^{2}\left(t\right)|u_{h}{|}^{2}\left|\right|u_{h}{\left|\right|}^{2}{\left|\eta_{h}\right|}^{2},\\ \tau^{2}\left(t\right)\left|b\left(u,\xi_{h},\eta_{h}\right)\right|\leq & \tau^{2}\left(t\right)(c_{1}^{2}{\left||u|\right|}^{2}|\xi_{h}\left|\;|\right|\xi_{h}\left|\right|+c_{1}^{2}\left||u||\;|\right|\xi_{h}{\left|\right|}^{2}\\ \; & +\frac{\nu }{8}\left|\right|\eta_{h}{\left|\right|}^{2}+\frac{1}{\nu }\left(\right|\eta_{h}{|}^{2}+{\left|u\right|}^{2}|\eta_{h}{|}^{2})), \end{array}$$

which implies, by using Lemma 6, that

(79) $$\begin{array}{cc} \; & \tau^{2}\left(t\right)|\eta_{h}{\left(t\right)|}^{2}+\int_{0}^{t}\tau^{2}\left(\tilde{t}\right)\left|\right|\eta_{h}{\left|\right|}^{2}d\tilde{t}\\ \leq & c_{1}^{2}\int_{0}^{t}\tau^{2}\left(\tilde{t}\right)\left(\left|\right|u_{h}{\left|\right|}^{2}|\xi_{h}\left|\;|\right|\xi_{h}\left|\right|+{\left||u|\right|}^{2}|\xi_{h}{|}^{1/2}\left|\right|\xi_{h}\left|\right|+\left|\right|u_{h}\left||\;|\right|\xi_{h}{\left|\right|}^{2}+\left||u||\;|\right|\xi_{h}{\left|\right|}^{2}\right)d\tilde{t}\\ \; & +\int_{0}^{t}\left[\frac{2}{\nu }\left(1+\kappa \right)+\left(\frac{2}{\nu }c_{1}^{2}+{\left(\frac{4}{\nu }\right)}^{4}c_{1}^{4}\kappa \right)\left|\right|u_{h}{\left|\right|}^{2})\right]\tau^{2}\left(\tilde{t}\right){\left|\eta_{h}\right|}^{2}d\tilde{t}+ch^{2}. \end{array}$$

Applying Theorems 1 and 2, Lemma 4, the H$\ddot{o}$lder inequality, and the inverse inequality (26), it holds true that

$$\begin{array}{cc} \; & c_{1}^{2}\int_{0}^{t}\tau^{2}\left(\tilde{t}\right)\left(\left|\right|u_{h}{\left|\right|}^{2}|\xi_{h}\left|\;|\right|\xi_{h}\left|\right|+{\left||u|\right|}^{2}|\xi_{h}\left|\;|\right|\xi_{h}\left|\right|\right)d\tilde{t}\\ \leq & \kappa c_{1}^{2}\int_{0}^{t}\tau^{2}\left(\tilde{t}\right)|\xi_{h}\left|\;|\right|\xi_{h}\left|\right|d\tilde{t}\\ \leq & \kappa c_{1}^{2}{\left(\int_{0}^{t}\tau^{2}\left(\tilde{t}\right){\left|\xi_{h}\right|}^{2}d\tilde{t}\right)}^{1/2}{\left(\int_{0}^{t}\tau^{2}\left(\tilde{t}\right)\left|\right|\xi_{h}{\left|\right|}^{2}d\tilde{t}\right)}^{1/2}\;\left(\mathrm{Cauchy}--\mathrm{Bunyakovsky}--\mathrm{Schwarz}\;\mathrm{inequality}\right)\\ \leq & ch^{4},\\ \; & c_{1}^{2}\int_{0}^{t}\tau^{2}\left(\tilde{t}\right)\left(\left|\right|u_{h}\left||\;|\right|\xi_{h}{\left|\right|}^{2}+\left||u||\;|\right|\xi_{h}{\left|\right|}^{2}\right)d\tilde{t}\\ \leq & \kappa c_{1}^{2}\int_{0}^{t}\tau^{3/2}\left(\tilde{t}\right)\sqrt{c_{2}}h^{-1/2}|\xi_{h}{|}^{1/2}\left|\right|\xi_{h}{\left|\right|}^{3/2}d\tilde{t}\;\left(\mathrm{inverse}\;\mathrm{inequality}\right)\\ \leq & \kappa c_{1}^{2}\sqrt{c_{2}}\int_{0}^{t}h^{1/2}\tau \left(\tilde{t}\right)\left|\right|\xi_{h}{\left|\right|}^{3/2}d\tilde{t}\\ \leq & \kappa c_{1}^{2}\sqrt{c_{2}}\sqrt[{4}]{T}{\left(\int_{0}^{t}\tau^{2}\left(\tilde{t}\right)\left|\right|\xi_{h}{\left|\right|}^{2}d\tilde{t}\right)}^{3/4}\;\left(H\ddot{o}\mathrm{lder}\;\mathrm{inequality}\right)\\ \leq & ch^{3}. \end{array}$$

Inputting the above estimates into (79) and using the Gronwall lemma yields

(80) $$\begin{array}{cc} \; & \tau^{2}\left(t\right)|\eta_{h}{\left(t\right)|}^{2}+\int_{0}^{t}\tau^{2}\left(\tilde{t}\right)\left|\right|\eta_{h}{\left|\right|}^{2}d\tilde{t}\leq ce^{M_{1}}h^{2}, \end{array}$$

where

$$\begin{array}{c} M_{1}=\int_{0}^{t}\left[\frac{2}{\nu }\left(1+\kappa \right)+\left(\frac{2}{\nu }c_{1}^{2}+{\left(\frac{4}{\nu }\right)}^{4}c_{1}^{4}\kappa \right)\left|\right|u_{h}{\left|\right|}^{2}\right]d\tilde{t}. \end{array}$$

Since

$$\begin{array}{cc} \; & e^{\int_{0}^{t}\left[\frac{2}{\nu }\left(1+\kappa \right)+\left(\frac{2}{\nu }c_{1}^{2}+{\left(\frac{4}{\nu }\right)}^{4}c_{1}^{4}\kappa \right)\left|\right|u_{h}{\left|\right|}^{2}\right]d\tilde{t}}\\ = & e^{\frac{2}{\nu }\left(1+\kappa \right)t+\left(\frac{2}{\nu }c_{1}^{2}+{\left(\frac{4}{\nu }\right)}^{4}c_{1}^{4}\kappa \right)e^{\int_{0}^{t}\left|\right|u_{h}{\left|\right|}^{2}d\tilde{t}}}\leq c, \end{array}$$

when inputting the above inequality into (80), (76) is followed. On the other hand, noting that τ(t)=1 for t≥1 and using Lemma 5, it is easy to check that (76) holds on (T,+∞).

Setting $v=e^{2\delta_{0}t}\tau^{3}\left(t\right)\eta_{ht}$ in (67), we obtain

(81) $$\begin{array}{cc} \; & e^{2\delta_{0}t}\tau^{3}\left(t\right)|\eta_{ht}{|}^{2}+\frac{\nu }{2}\frac{d}{dt}(e^{2\delta_{0}t}\tau^{3}\left(t\right)||\eta_{h}{\left|\right|}^{2})\\ = & \nu \left(\frac{3}{2}+\delta_{0}\right)e^{2\delta_{0}t}\tau^{2}\left(t\right)\left|\right|\eta_{h}{\left|\right|}^{2}-e^{2\delta_{0}t}\tau^{3}\left(t\right)\left[b\left(e_{h},u_{h},\eta_{ht}\right)+b\left(u,e_{h},\eta_{ht}\right)\right]. \end{array}$$

Due to (7), there hold that

$$\begin{array}{cc} \tau^{3}\left(t\right)\left|b\left(\xi_{h},u_{h},\eta_{ht}\right)\right|\leq & c_{1}\tau^{3}\left(t\right)(|\xi_{h}{|}^{1/2}\left|\right|\xi_{h}{\left|\right|}^{1/2}\left|\right|u_{h}{\left|\right|}^{1/2}{\left|A_{h}u_{h}\right|}^{1/2}\\ \; & +\left|\right|\xi_{h}\left||\;\right|u_{h}{|}^{1/2}|A_{h}u_{h}{|}^{1/2})|\eta_{ht}|\\ \leq & \frac{1}{4}\tau^{3}\left(t\right)|\eta_{ht}{|}^{2}+2c_{1}^{2}\tau^{3}\left(t\right)|\xi_{h}\left|\;|\right|\xi_{h}\left||\;|\right|u_{h}\left||\;\right|A_{h}u_{h}|\\ \; & +2c_{1}^{2}\tau^{3}\left(t\right)||\xi_{h}{\left|\right|}^{2}|u_{h}\left|\;\right|A_{h}u_{h}|,\\ \tau^{3}\left(t\right)\left|b\left(\eta_{h},u_{h},\eta_{ht}\right)\right|\leq & \frac{1}{4}\tau^{3}\left(t\right)|\eta_{ht}{|}^{2}+2c_{1}^{2}\tau^{3}\left(t\right)|\eta_{h}\left|\;|\right|\eta_{h}\left||\;|\right|u_{h}\left||\;\right|A_{h}u_{h}|\\ \; & +2c_{1}^{2}\tau^{3}\left(t\right)||\eta_{h}{\left|\right|}^{2}|u_{h}\left|\;\right|A_{h}u_{h}|,\\ \tau^{3}\left(t\right)\left|b\left(u,\xi_{h},\eta_{ht}\right)\right|\leq & c_{1}\tau^{3}{\left(t\right)(|u|}^{1/2}{\left|Au\right|}^{1/2}\left|\right|\xi_{h}\left|\right|+{\left||u|\right|}^{1/2}{\left|Au\right|}^{1/2}|\xi_{h}{|}^{1/2}\left|\right|\xi_{h}{\left|\right|}^{1/2})|\eta_{ht}|\\ \leq & \frac{1}{8}\tau^{3}\left(t\right)|\eta_{ht}{|}^{2}+4c_{1}^{2}\tau^{3}\left(t\right)|u|\;|Au|\;||\xi_{h}{\left|\right|}^{2}\\ \; & +4c_{1}^{2}\tau^{3}\left(t\right)||u||\;|Au|\;|\xi_{h}\left|\;|\right|\xi_{h}\left|\right|,\\ \tau^{3}\left(t\right)\left|b\left(u,\eta_{h},\eta_{ht}\right)\right|\leq & \frac{1}{8}\tau^{3}\left(t\right)|\eta_{ht}{|}^{2}+4c_{1}^{2}\tau^{3}\left(t\right)|u|\;|Au|\;||\eta_{h}{\left|\right|}^{2}\\ \; & +4c_{1}^{2}\tau^{3}\left(t\right)||u||\;|Au|\;|\eta_{h}\left|\;|\right|\eta_{h}\left|\right|, \end{array}$$

Combining these estimates with (81), integrating from 0 to *t*, using the H$\ddot{o}$lder inequality, and multiplying by $e^{-2\delta_{0}t}$, we arrive at

(82) $$\begin{array}{cc} \tau^{3}\left(t\right)||\eta_{h}{\left(t\right)||}^{2}+e^{-2\delta_{0}t}\int_{0}^{t}e^{2\delta_{0}\tilde{t}}\tau^{3}\left(\tilde{t}\right){\left|\eta_{h\tilde{t}}\right|}^{2}d\tilde{t}\leq & ch^{2}. \end{array}$$

The proof is completed. □

**Theorem** **3.** *Under the assumptions of Lemma 5, we have, for all t>0,*

(83) $$\begin{array}{cc} e^{-2\delta_{0}t}\int_{0}^{t}e^{2\delta_{0}\tilde{t}}\tau \left(\tilde{t}\right){\left|u-u_{h}\right|}^{2}d\tilde{t}\leq & ch^{2}, \end{array}$$

(84) $$\begin{array}{cc} \tau^{2}\left(t\right)|u\left(t\right)-u_{h}{\left(t\right)|}^{2}+e^{-2\delta_{0}\tilde{t}}\int_{0}^{t}e^{2\delta_{0}\tilde{t}}\tau^{2}\left(\tilde{t}\right)||u-u_{h}{\left|\right|}^{2}d\tilde{t}\leq & ch^{2}, \end{array}$$

(85) $$\begin{array}{cc} \tau^{3}\left(t\right)||u\left(t\right)-u_{h}{\left(t\right)||}^{2}+e^{-2\delta_{0}t}\int_{0}^{t}e^{2\delta_{0}\tilde{t}}\tau^{3}\left(\tilde{t}\right){\left|u_{\tilde{t}}-u_{h\tilde{t}}\right|}^{2}d\tilde{t}\leq & ch^{2}, \end{array}$$

(86) $$\begin{array}{cc} \tau^{4}\left(t\right){\left|p\left(t\right)-p_{h}\left(t\right)\right|}^{2}\leq & ch^{2}. \end{array}$$

**Proof.**  By using Lemmas 4 and 6, we have (83)–(85). To prove (86), subtracting (31) from (10), we arrive at

(87) $$\begin{array}{c} \left(e_{ht},v\right)+a\left(e_{h},v\right)-d\left(v,p\right)+b\left(e_{h},u_{h},v\right)+b\left(u,e_{h},v\right)=0,\;\;\;\forall v\in V_{h}. \end{array}$$

From the definition of $P_{h}$ and (87), there holds that

(88) $$\begin{array}{cc} (e_{ht},v)= & \left(e_{ht},\left(v-P_{h}v\right)\right)+\left(e_{ht},P_{h}v\right)\\ = & \left(e_{ht},\left(v-P_{h}v\right)\right)-a\left(e_{h},P_{h}v\right)\\ \; & +d\left(P_{h}v,p\right)-b\left(e_{h},u_{h},P_{h}v\right)-b\left(u,e_{h},P_{h}v\right). \end{array}$$

By (6), (28) and (29), we have

$$\begin{array}{cc} |d(P_{h}v,p)|= & |\left(p,\nabla \cdot P_{h}v\right)\left|=\right|\left(p-J_{h}p,\nabla \cdot P_{h}v\right){\left|\leq ch||p|\right|}_{1}||v||,\\ |b(e_{h},u_{h},P_{h}v)|\leq & c_{1}\left(\right|e_{h}{|}^{1/2}\left|\right|e_{h}{\left|\right|}^{1/2}\left|\right|u_{h}\left|\right|+\left|\right|e_{h}\left||\;\right|u_{h}{|}^{1/2}\left|\right|u_{h}{\left|\right|}^{1/2})||v||,\\ |b(u,e_{h},P_{h}v)|\leq & c_{1}{\left(|u\right|}^{1/2}{\left||u|\right|}^{1/2}\left|\right|e_{h}\left|\right|+\left||u||\;\right|e_{h}{|}^{1/2}\left|\right|e_{h}{\left|\right|}^{1/2})||v||,\\ |(e_{ht},v-P_{h}v)|\leq & ch(|u_{t}|+|u_{ht}|)||v||,\\ |a(e_{h},P_{h}v)|\leq & c||e_{h}||\;||v||. \end{array}$$

Taking these estimates into (88), and using Theorems 1 and 2, (84) and (85), we obtain

(89) $$\begin{array}{cc} \tau^{2}\left(t\right)||e_{ht}{\left|\right|}_{-1}= & \tau^{2}\left(t\right)\underset{v\in V_{h},v\neq 0}{\sup}\frac{\left(e_{ht},v\right)}{\left||v|\right|}\\ \leq & ch+\tau^{2}\left(t\right)||e_{h}\left|\right|+c_{1}^{2}\tau^{3/2}\left(t\right)|e_{h}{|}^{1/2}\left|\right|e_{h}{\left|\right|}^{1/2}+c_{1}^{2}\tau^{3/2}\left(t\right)||e_{h}\left|\right|\\ \leq & ch. \end{array}$$

Due to the discrete LBB condition and applying a similar process to that in (89), there holds

(90) $$\begin{array}{cc} \tau^{4}\left(t\right){\left|p-p_{h}\right|}^{2}\leq & c(\tau^{4}\left(t\right)||e_{ht}|{|}_{-1}^{2}+\tau^{4}\left(t\right)||e_{h}|{|}^{2}+c_{1}^{2}\tau^{3}\left(t\right)|e_{h}|\;||e_{h}||+c_{1}^{2}\tau^{3}\left(t\right)|e_{h}|\;||e_{h}||)\\ \leq & ch^{2}. \end{array}$$

The proof is completed. □

## 5. Numerical Examples

In this section, we show some numerical examples to verify the theoretical prediction. Taking $f\left(x,t\right)={\left(10\cos\left(1000\pi t\right),10\cos\left(1000\pi t\right)\right)}^{T}$, ν=10, Ω=(0,1)×(0,1) and the time step Δt=1/20000 (the implicit Euler scheme is applied to the temporal discretization), and using mini-element in the spatial approximation, we investigate the solutions $\left(u_{h}^{n},p_{h}^{n}\right)$ with different nonsmooth initial data.

**Figure 1:**
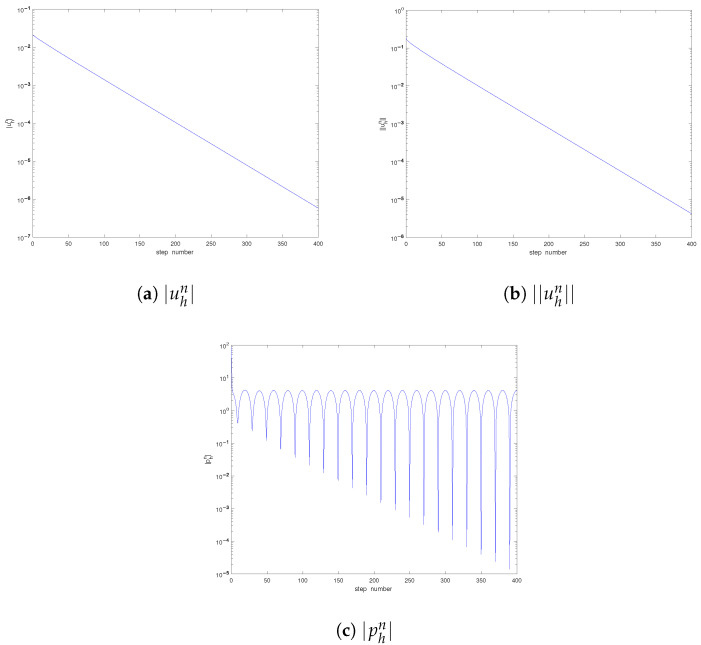
Development of the solution (Case I).

**Case I**: Setting

$$u_{1}\left(x_{1},x_{2},0\right)=\left\{\begin{array}{c} 10x_{1}^{2}{\left(x_{1}-1\right)}^{2}x_{2}\left(x_{2}-1\right)\left(2x_{2}-1\right),\;x_{1}\geq 0.5,\\ 0,\;x_{1}<0.5, \end{array}\right.$$

$$\;u_{2}\left(x_{1},x_{2},0\right)=\left\{\begin{array}{c} -10x_{1}\left(x_{1}-1\right)\left(2x_{1}-1\right)x_{2}^{2}{\left(x_{2}-1\right)}^{2},\;x_{1}\geq 0.5,\\ 0,\;x_{1}<0.5, \end{array}\right.$$

it is easily to check that $u_{0}\left(x\right)={\left(u_{1}\left(x_{1},x_{2},0\right),u_{2}\left(x_{1},x_{2},0\right)\right)}^{T}$, satisfying $\nabla \cdot u_{0}=0$ and $u_{0}\in L^{2}\left(\Omega \right)$. Under the computational environment set above, using the numerical solutions obtained with h=1/100 as the “reference solutions” (denoted by $\left(u_{ref},p_{ref}\right)$), we first study the convergence order of the spatial discretization in Table 1, Table 2 and Table 3. From the results, we can find that, despite the existence of the singularity of the solutions near t=0, the predicted convergence orders are almost achieved for all tested cases. Moreover, as the time tends to 0 (from the 10th step to the 2nd step), all corresponding errors uniformly increase. Then, we study the developments of the solutions in Figure 1, which suggests that the values of $|u_{h}^{n}|$, $\left|\right|u_{h}^{n}\left|\right|$, and $|p_{h}^{n}|$ all increase rapidly as the time decreases. As the time develops, the pressure will arrive at a relative steady state and have the same period with respect to the time as that of the body force |f(x,t)| (see Figure 1c); all of these are consistent with the theoretical predictions.

To further confirm the theoretical deduction, we consider two other cases with nonsmooth initial data.

**Case II**:

$$u_{1}\left(x_{1},x_{2},0\right)=\left\{\begin{array}{c} 2\pi {\left(\sin\left(\pi x_{1}\right)\right)}^{2}\sin\left(\pi x_{2}\right)\cos\left(\pi x_{2}\right),\;\;\;\;\;\;\;x_{1}\geq 0.5,\\ 0,\;x_{1}<0.5, \end{array}\right.$$

$$\;u_{2}\left(x_{1},x_{2},0\right)=\left\{\begin{array}{c} -2\pi \sin\left(\pi x_{2}\right)\cos\left(\pi x_{1}\right){\left(\sin\left(\pi x_{2}\right)\right)}^{2},\;x_{1}\geq 0.5,\\ 0,\;x_{1}<0.5, \end{array}\right.$$

**Case III** [13]:

$$\begin{array}{cc} u_{1}\left(x_{1},x_{2},0\right)= & 1.5\pi {\left(\sin\left(\pi x_{1}\right)\right)}^{1.5}{\left(\sin\left(\pi x_{2}\right)\right)}^{0.5}\cos\left(\pi x_{2}\right),\\ u_{2}\left(x_{1},x_{2},0\right)= & -1.5\pi {\left(\sin\left(\pi x_{1}\right)\right)}^{0.5}\cos\left(\pi x_{1}\right){\left(\sin\left(\pi x_{2}\right)\right)}^{1.5}. \end{array}$$

**Figure 2:**
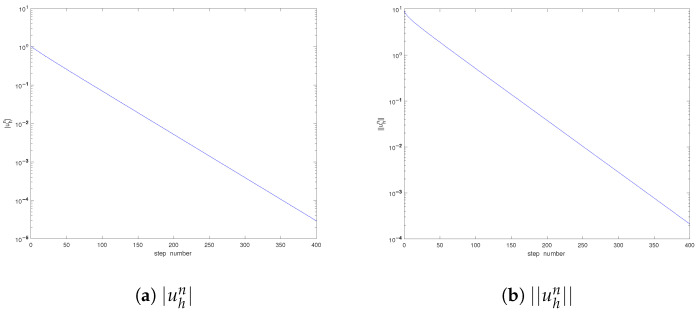

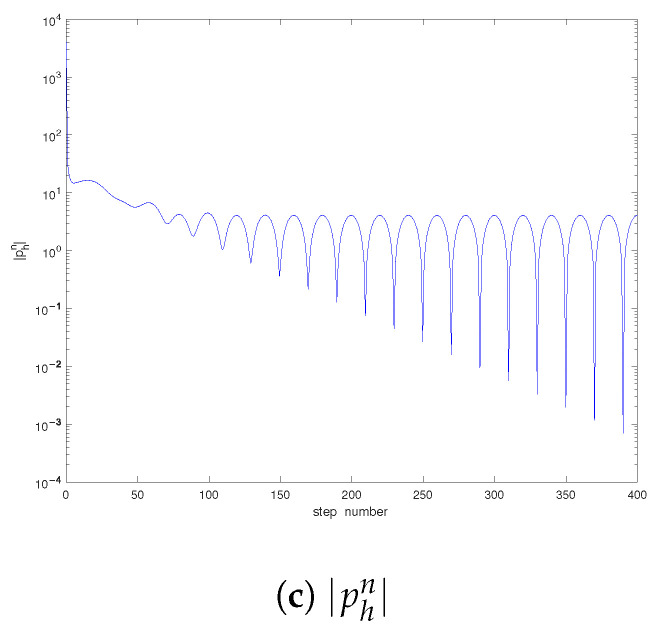
Development of the solution (Case II).

**Figure 3:**
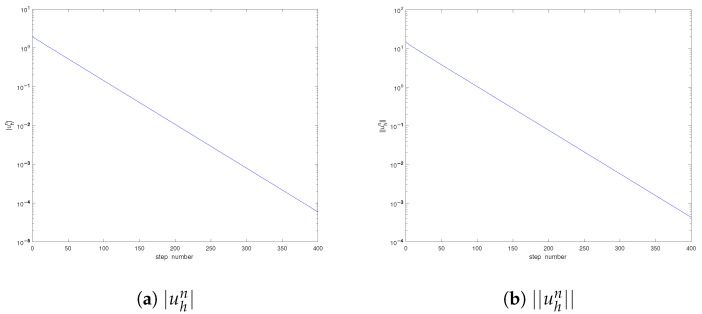

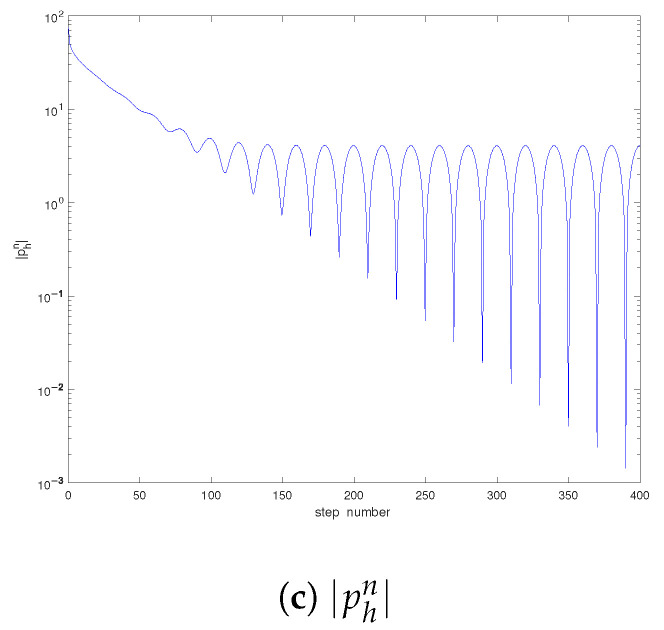
Development of the solution (Case III).

These two initial data also belong to $L^{2}\left(\Omega \right)$ and satisfy the incompressibility condition. With the same computational parameters as above, we show the convergence orders in Table 4, Table 5, Table 6, Table 7, Table 8 and Table 9 and the developments of the solutions in Figure 2 and Figure 3. Similar phenomena can be observed. Again, the singularity of the solution is confirmed. Furthermore, we can find that the times when the pressure periods begin are different in Figure 1, Figure 2 and Figure 3; the reason is that it also depends on the initial data. On the other hand, as the time developed becomes large enough, the period for the velocity *u* will appear too, which is omitted here since we are interested in the singularity near t=0.

## 6. Conclusions

In this paper, we analyzed the finite element error estimate for the Navier–Stokes equations with $L^{2}$ initial data. By introducing an intermediate step and using the integral techniques and dual-norm estimate, we derived the finite element bounds for the velocity and pressure. However, due to the singularity of the solution on t∈[0,1), we did not obtain the optimal error estimate for the velocity in $L^{2}$-norm. Moreover, only the error estimates for the spatial semi-discrete finite element method were derived. How does the technique in this paper extend to the fully discrete scheme, especially with a higher order scheme (see, e.g., [23])? All of these will be considered in our further work.

## Figures and Tables

**Table 1:** Absolute errors and convergence orders at the 2nd time step (Case I).

*h*	$|p_{ref}-p_{h}^{n}|$	Rate	$|u_{ref}-u_{h}^{n}|$	Rate	$\left|\right|u_{ref}-u_{h}^{n}\left|\right|$	Rate
1/10	0.330028	−	0.001208	−	0.051935	−
1/20	0.158543	1.06	0.000349	1.79	0.029132	0.83
1/30	0.086556	1.49	0.000153	2.04	0.019055	1.05
1/40	0.051528	1.80	0.0000818	2.17	0.015250	0.77

**Table 2:** Absolute errors and convergence orders at the 5th time step (Case I).

*h*	$|p_{ref}-p_{h}^{n}|$	Rate	$|u_{ref}-u_{h}^{n}|$	Rate	$\left|\right|u_{ref}-u_{h}^{n}\left|\right|$	Rate
1/10	0.222297	−	0.001006	−	0.038672	−
1/20	0.064695	1.78	0.000256	1.98	0.020591	0.91
1/30	0.031808	1.75	0.000111	2.06	0.013587	1.03
1/40	0.020123	1.59	0.0000591	2.19	0.010815	0.79

**Table 3:** Absolute errors and convergence orders at the 10th time step (Case I).

*h*	$|p_{ref}-p_{h}^{n}|$	Rate	$|u_{ref}-u_{h}^{n}|$	Rate	$\left|\right|u_{ref}-u_{h}^{n}\left|\right|$	Rate
1/10	0.160356	−	0.000938	−	0.030927	−
1/20	0.050491	1.67	0.000233	2.01	0.016439	0.91
1/30	0.025932	1.64	0.000100	2.09	0.010826	1.03
1/40	0.016863	1.50	0.0000528	2.22	0.008629	0.79

**Table 4:** Absolute errors and convergence orders at the 2nd time step (Case II).

*h*	$|p_{ref}-p_{h}^{n}|$	Rate	$|u_{ref}-u_{h}^{n}|$	Rate	$\left|\right|u_{ref}-u_{h}^{n}\left|\right|$	Rate
1/10	15.1954	−	0.0651914	−	2.75097	−
1/20	7.59238	1.00	0.018392	1.83	1.53808	0.84
1/30	4.12981	1.50	0.008021	2.05	1.00779	1.04
1/40	2.45499	1.81	0.004290	2.18	0.80603	0.78

**Table 5:** Absolute errors and convergence orders at the 5th time step (Case II).

*h*	$|p_{ref}-p_{h}^{n}|$	Rate	$|u_{ref}-u_{h}^{n}|$	Rate	$\left|\right|u_{ref}-u_{h}^{n}\left|\right|$	Rate
1/10	10.0955	−	0.053100	−	1.96004	−
1/20	2.84993	1.82	0.013678	1.96	1.05254	0.90
1/30	1.38272	1.78	0.005949	2.05	0.695929	1.02
1/40	0.866596	1.62	0.003160	2.20	0.554039	0.79

**Table 6:** Absolute errors and convergence orders at the 10th time step (Case II).

*h*	$|p_{ref}-p_{h}^{n}|$	Rate	$|u_{ref}-u_{h}^{n}|$	Rate	$\left|\right|u_{ref}-u_{h}^{n}\left|\right|$	Rate
1/10	7.32485	−	0.0473821	−	1.51272	−
1/20	2.24572	1.71	0.0120436	1.98	0.807485	0.91
1/30	1.14128	1.67	0.0051888	2.08	0.532222	1.03
1/40	0.73624	1.52	0.0027371	2.22	0.424337	0.79

**Table 7:** Absolute errors and convergence orders at the 2nd time step (Case III).

*h*	$|p_{ref}-p_{h}^{n}|$	Rate	$|u_{ref}-u_{h}^{n}|$	Rate	$\left|\right|u_{ref}-u_{h}^{n}\left|\right|$	Rate
1/10	27.3182	−	0.106307	−	4.35144	−
1/20	8.21586	1.73	0.027245	1.96	2.23334	0.96
1/30	4.23649	1.63	0.011775	2.07	1.46822	1.03
1/40	2.79604	1.44	0.0062589	2.20	1.15596	0.83

**Table 8:** Absolute errors and convergence orders at the 5th time step (Case III).

*h*	$|p_{ref}-p_{h}^{n}|$	Rate	$|u_{ref}-u_{h}^{n}|$	Rate	$\left|\right|u_{ref}-u_{h}^{n}\left|\right|$	Rate
1/10	19.3230	−	0.0877984	−	3.34969	−
1/20	6.16618	1.65	0.0213474	2.04	1.73706	0.95
1/30	3.22885	1.60	0.0091224	2.10	1.14311	1.03
1/40	2.13412	1.44	0.0048206	2.22	0.90731	0.80

**Table 9:** Absolute errors and convergence orders at the 10th time step (Case III).

*h*	$|p_{ref}-p_{h}^{n}|$	Rate	$|u_{ref}-u_{h}^{n}|$	Rate	$\left|\right|u_{ref}-u_{h}^{n}\left|\right|$	Rate
1/10	14.7695	−	0.0801361	−	2.72252	−
1/20	4.87391	1.60	0.0192647	2.06	1.42985	0.92
1/30	2.56145	1.59	0.0081812	2.11	0.93953	1.04
1/40	1.69241	1.44	0.0043027	2.23	0.74845	0.79

## Data Availability

Not applicable.
